# Expression and differential distribution of the shaker-related voltage-gated potassium channel family (K_V_1.x) in human hippocampus and neocortex

**DOI:** 10.1186/1471-2210-11-S2-A27

**Published:** 2011-09-05

**Authors:** Irmgard Leitner, Georg Wietzorrek, Maria Trieb, Josef Marksteiner, Hans-Günther Knaus

**Affiliations:** 1 Division of Molecular and Cellular Pharmacology, Medical University Innsbruck, 6020 Innsbruck, Austria; 2 Psychiatric Hospital Hall, 6060 Hall in Tirol, Austria

## Background

All excitable cells express voltage-gated potassium (K_V_) channels; in neurons they play an essential role in setting the resting membrane potential, controlling the firing frequency and duration of action potentials, and modulate neurotransmitter release. Due to this critical function as regulators of neuronal excitability, mutations and/or deletions in potassium channel subunit genes are associated with diverse clinical phenotypes (channelopathies), including seizure or movement disorders in both humans and animals. Additionally, voltage-gated potassium channels might play a crucial role in neurodegenerative and psychiatric disorders. For this reason, a better understanding of the occurrence and specific distribution of voltage-gated potassium channels would be highly necessary. Several members of the K_V_1 subfamily have been found, but only K_V_1.1, K_V_1.2, K_V_1.4 and K_V_1.6 are widely expressed in the CNS in both human and rodent brain. However, unlike to rodents, little is known regarding the regional localization of these four members of the K_V_1 subfamily in human brain. Therefore we investigated, for the first time, the distribution of these four K_V_1 channel subtypes in human neocortex and hippocampus, which are known for their vulnerability to epilepsy and their importance for learning, memory and cognitive processes.

## Methods and results

To examine the relative expression levels of the K_V_1.1, K_V_1.2, K_V_1.4 and K_V_1.6 proteins in human as well in mouse brain and to determine the specificity of each individual α-subunit antibody for human brain tissue, Western blots were conducted. Individual expression patterns for each K_V_1 channel subtype were established by immunohistochemistry using polyclonal anti-K_V_1.1, KV1.2, K_V_1.4, and K_V_1.6 as primary antibodies. We found that the staining patterns of these four K_V_1 channel subunits overlap in some areas but each K_V_ channel subunit shows a unique pattern of distribution in human cortex and hippocampus. The pyramidal cell bodies of cornu Ammonis (CA) 1–3 areas and the granule cell bodies of the dentate gyrus were strongly immunoreactive for K_V_1.1, K_V_1.2, K_V_1.4 and K_V_1.6. Varying degrees of immunoreactivity were also found in other layers, such the inner and outer molecular layer, stratum lacunosum and stratum oriens.

## Conclusions

Precise knowledge of the differential distribution of K_V_1 channels in human brain may provide useful data for future investigations on common pathological conditions such as epilepsy and neurodegenerative disorders.

